# Opioid-induced immunosuppression and carcinogenesis promotion theories create the newest trend in acute and chronic pain pharmacotherapy

**DOI:** 10.6061/clinics/2020/e1554

**Published:** 2020-03-16

**Authors:** Urszula Kosciuczuk, Pawel Knapp, Anna Maria Lotowska-Cwiklewska

**Affiliations:** I Department of Anesthesiology and Intensive Therapy, Medical University of Bialystok, Department of Anesthesiology and Intensive TherapyMedical University of BialystokPolandPoland; II Department of Gynecology and Gynecological Oncology, Medical University of Bialystok, Department of Gynecology and Gynecological OncologyMedical University of BialystokPolandPoland.

**Keywords:** Immunity, Cancer, Opioids, Oxidative Stress, Pain

## Abstract

Opioids are the main group of pharmacological agents used during the perioperative period and provide a sedative and analgesic component. The observations of opioid consumption in West Europe indicate that this group of drugs is widely used in chronic noncancer pain therapy.

Nearly 20 years ago, the first publications indicating that opioids, as an element of perioperative pharmacotherapy in oncologic patients, increase the risk of tumor recurrence and affect further prognosis were presented. The actual publications suggest that there are multifactorial, complex mechanisms underlying the immunological impact and carcinogenesis promotion of opioids and that the intensity varies depending on the type of opioid. There are also questions about the immunosuppressive effects among patients receiving opioids in the treatment of chronic noncancer pain.

The aim of the review article is to present information about the action of opioids on the immune system in carcinogenic settings and to define the clinical usefulness of this pharmacological phenomenon.

## INTRODUCTION

Opioids are the main group of pharmacological agents used during general anesthesia and provide a sedative and analgesic component. In addition to these two effects, it should also be emphasized that these substances cause an immunosuppressive effect and exacerbate this effect in the perioperative period. Opioid-induced immunosuppression, especially in oncologic patients, is a hot topic of current perioperative pharmacology research. This is a new trend in complex oncologic treatment: not only does the surgical resection of the lesion begin the therapeutic process, but the whole perioperative period, including the pharmacological aspects of the method of anesthesia and postoperative analgesia, is of great importance (1,2).

Nearly 20 years ago, the first publications indicating that opioids affect prognosis in oncologic therapy were presented. Many authors demonstrated a reduced risk of tumor recurrence connected with the use of regional anesthetic techniques and limitation of opioid use in the perioperative period. It has also been suggested that the intensity of these effects varies depending on the type of opioid. This is particularly important in cancer-related pain and in the perioperative period, especially in patients with early-stage cancer. The observations of opioid consumption in West Europe indicate that this group of drugs is widely used in chronic noncancer pain therapy. There are also questions about the effects of the immunosuppressive mechanisms among patients receiving opioids in the treatment of chronic noncancer pain. The influence of opioids on the immune system and carcinogenesis has not been fully explained. In view of the increasing use of opioid drugs and their important place in multimodal oncologic therapy, the connection of opioids with the immune system and cancer has a crucial role (3,4).

It has also been suggested that the intensity of immunosuppressive effects varies depending on the type of opioid. It was also described that in many tumor tissues, increased expression of opioid receptors was observed, and their accumulation was also associated with an increased risk of cancer progression with metastasis. The presented reports are a very important element of multidirectional cancer therapy. With respect to such publications, the question should be asked as to whether to limit the use of opioid drugs in oncologic patients (3-5).

The aim is to present information about the mechanisms underlying the influence of opioid drugs on the immune system in a cancer setting and determine the clinical usefulness of this phenomenon in multimodal oncologic treatment.

### Opioid receptors

The first publications regarding the location of opioid receptors and the endogenous opioid peptide system in the 1970s and 1980s indicated that these substances were associated with the nervous system. However, subsequent experimental studies have indicated that the opioid receptor system is widely distributed in both peripheral and central nervous system structures, in structures of ascending (peripheral nerves, the spinal cord dorsal horn, the brain stem, the thalamus, and the cortex) and descending pain transmission pathways, in organs of the autonomic nervous system and in the immune system. Recent publications indicate that opioid receptors are also found in tissues involved in the inflammatory process as well as in cancerous tissues (5,6).

Opioid receptors are classified into the following groups: the classic naloxone-sensitive receptors, mi opioid receptor (MOR), delta opioid receptor (DOR), and kappa opioid receptor (KOR), and nonclassic naloxone-insensitive receptors (NORs). Receptor-specific interactions induce analgesic effects; however, many authors have emphasized that only MORs are associated with the effects leading to respiratory depressive, tolerance-inducing and immunosuppressive effects (7-10).

The effects of immunomodulation have been related to interactions with MORs and NORs. The functions and significance of MORs have been well understood and described. The most important aspect related to NOR activation is analgesic effects at the spinal cord level, and unlike other opioid receptors, NORs do not contribute to the development of respiratory depression and have minimal risk of tolerance or addiction (11-14).

### Opioids, immunity, and oxidative stress

In terms of elements of innate immunity, the activation of opioid receptors results in a decrease in the number of macrophages, reduced leukocyte migration to inflammatory sites, decreased phagocytotic activity of macrophages, decreased chemotaxis and superoxide production by neutrophils and macrophages, accelerated apoptosis of macrophages, weakened microbiological protection, and reduced adhesion of leukocytes and endothelial cells. Other negative effects have also been described in relation to mast cells, dendritic cells, and natural killer (NK) cells. Opioids impair intestinal barrier function and promote systemic infections by increasing the sensitivity of gut epithelial cells to Toll-like receptor activation with bacterial translocation. In addition, opioid treatment changes the gut microbial composition and induces the expansion of gram-positive pathogens. These mechanisms promote the translocation of harmful pathogens and result in the risk of systemic infection (6-8,12). Regarding the elements of adaptive immunity, proliferative and secretory hyperactivity of T helper cells (Th), increased apoptosis of the subpopulation of Th1 cells with increased differentiation of the subpopulation Th 2 cells, a decreased Th1/Th2 ratio, a decreased CD4/CD8 Th ratio, and decreased NK activity have been reported. In humoral aspects, lower concentrations of interleukin 1 beta, interleukin 2, tumor necrosis factor alpha and interferon-gamma were described. Regarding B cells, decreased production of antibodies, decreased activity in the expression of tissue compatibility factors and proliferation, decreased mitotic response after stimulation with bacterial antigen, increased secretion of transforming growth factor beta, and other anti-inflammatory cytokines and increased T cell apoptosis due to nuclear factor kappa B and the activator protein 1/nuclear factor of activated T cells pathways have been observed. The indirect mechanism of the interaction of opioid drugs with the immune system has been associated with the neuroendocrine axis described as the hypothalamic-pituitary-adrenal (HPA) axis. Opioid receptor activation in central nervous system structures stimulates corticotropin-releasing hormone secretion, which activates the production of adrenocorticotropic hormone in the anterior pituitary region, with the final effect of producing glucocorticoids in the adrenal cortex. The catecholamines excreted from the adrenal glands showed depression of NK cell activity, as well as decreased proliferation and differentiation of peripheral blood lymphocytes. The effects associated with glucocorticosteroids are associated with the modulation of transcription of genes related to both innate and adaptive immune defense (13-17).

Other publications have also confirmed the multidirectional role opioids play in carcinogenesis by regulating the transcription of tumor growth factors and promoting capillary formation and neovascularization of cancerous tissue. It has been shown that the MORs regulate the proliferation and migration of non-small-cell lung cancer cells through interactions at the level of gene transcription: nonreceptor tyrosine kinase (Src), associated binding protein 1 (Gab-1/GRB 2), phosphatidylinositol-4,5-biphosphate 3-kinase catalytic subunit alpha (PI3CA), signal transducer and activator of transcription 3 (STAT 3), serine/threonine kinase 1 (Akt) (18,19). Connolly et al. analyzed the expression of genes connected with the analgesic effect of opioids in breast tumor tissue and found that these genes play an important role in cancer progression (20). The authors also identified a high level of MOR and DOR gene expression as conferring a high risk of metastasis. Increased expression of MORs in prostate tumor tissues or lung cancer was a risk factor for disease progression, unfavorable prognosis and increased demand for opioid drugs in the postoperative period (21,22).

Another mechanism of opioid-regulated carcinogenesis is connected with microRNA function (miRNA), which are endogenous factors, including noncoding RNAs, which regulate gene expression and posttranscriptional modification. It was described that opioids induce upregulation of miRNA 221 and the let-7 family, which are important factors in carcinogenesis and apoptosis, and downregulation of miRNA-92, which is crucial for proliferation, invasion, and migration (23,24).

The next mechanism of opioid-induced immunosuppression is connected with oxidative stress and reactive oxygen species (ROS) function. ROS are known as the most important mutagenic factor, causing DNA damage and contributing to malignant transformation. The ROS concentration is a final balance of oxidative and antioxidative processes. Their low stability and high diversity of reactions complicate their intracellular and fluid measurements, and many other biochemical markers have been described as predictors of oxidative balance (superoxide dismutase, catalase, glutathione peroxidase, and ascorbic acid). The newest laboratory measures are total oxidative capacity and total antioxidative capacity, which present a summary of all mechanisms (3,6).

### Clinical aspects

The opioid-induced immunosuppression phenomenon in anesthesia and acute postoperative pain is a current topic. Although no relevant recommendations have been developed yet, methods of low-dose opioid or even nonopioid anesthesia, multimodal perioperative analgesia with the use of local anesthetics and regional analgesia are performed. It seems that in the perioperative period, many pharmacological substances used during general anesthesia cause immunosuppression through modulation of the oxidative balance and activation of HPA and the sympathomimetic system. These mechanisms have been reported to increase ROS concentration and release catecholamines and prostaglandins, which in turn induce the secretion of chemokines, immunosuppressive cytokines and proinflammatory cytokines that promote angiogenesis in tumor tissues and metastases (12,14,16).

The clinical effects of opioid-induced immunosuppression have been described in many aspects. It was shown that opioid analgesia deprived the cytotoxic function of NK lymphocytes more than ketorolac did, and the immunosuppressive effect assessed by the cytotoxicity of NK lymphocytes was stronger after using morphine and had a smaller effect after using oxycodone than after using opioids (25,26). Combined general anesthesia based on opioid analgesia increased the expression of MORs in breast tumor tissues compared to total intravenous anesthesia with propofol and the technique of paravertebral analgesia with bupivacaine solutions (16).

The phenomenon of increased expression of opioid receptors in gynecologic cancers and the effect of opioid use in the perioperative period on prognosis have also been described. Levins et al. showed that MOR expression on breast cancer cells was higher in patients who underwent combined volatile anesthesia with perioperative opioid analgesia. Authors suggested that administration of propofol, a paravertebral anesthetic, without opioids decreased MOR expression. A retrospective analysis of the course of treatment of patients with ovarian cancer demonstrated that nonopioid analgesia, epidural anesthesia and the continuation of this method in the perioperative period may reduce mortality after 3 and 5 years of observation (16).

An important aspect of immunosuppression associated with the use of opioids in cancer patients is the risk of infection. Total daily opioid dose was an independent factor that influenced the development of infection in opioid monotherapy in cancer patients, and the risk for developing infection increased by 2% per 10 mg increase in an oral morphine equivalent (27).

Opioid therapy is also used as an element in pharmacotherapy for neuropathic pain, which very often persists as a consequence of chemotherapy, radiotherapy or cancer-related neuropathy. The pathogenesis of neuropathic pain points to multifactorial influences with a strongly expressed component of excessive immune activation. In these clinical situations, the opioid-induced immunosuppression phenomenon has a crucial and beneficial effect (10,15).

A separate issue is the risk of developing cancer in natural opioid users and among patients with chronic noncancer pain. Prolonged use of opium or its derivatives has been reported to be an important risk factor for oral cavity cancers, upper gastrointestinal tract cancers, pancreatic cancers, and bladder, kidney, larynx, pharynx and lung cancers (28-33).

The role of opioid administration on the human immune system and cancer progression cannot be unequivocally assessed. Despite many observational and experimental studies, these conclusions are contentious. A serious limitation of the assessment of the clinical usefulness is the biological diversity of tumors. Most publications presenting the topic of the influence of opioid drugs on the course of cancer are based on studies of gastrointestinal tract, lung, prostate and breast cancer. In connection with epidemiological data, it is extremely important to assess the data of opioid influence on the progression of the most frequently occurring and poorest prognosis cancers (34-37).

Opioid tolerance is another important clinical aspect of immunosuppression. Approximately 60% of patients develop drug tolerance during opioid therapy. However, the crucial mechanisms are not completely understood, and the role of microglia and astrocyte activation is the newest theory. A key point is that opioids have direct effects on non-neuronal cells, in particular, mast cells, astrocytes, and microglia. Mast cell activation releases numerous cytokines (tumor necrosis factor, interleukin-1, and tryptase) and pronociceptive substances, such as substance P, that contribute to the exacerbation of proinflammatory processes and nociception. Indeed, glial cell-produced chemokines decrease the expression of receptors to antinociceptive mediators (gamma-aminobutyric acid - GABA) and increase the number of pronociceptive mediators (alpha-amino-3-hydroxy-5-methyl-4-isoxazolepropionic acid – AMPA; and N-methyl-D-aspartate - NMDA), decrease glutamate transporter proteins, and decrease outward potassium currents. Many authors have shown that both acute and chronic opioid administration, due to microglia and astrocyte activation and increased levels of pronociceptive stimulation mediators, require an increased demand for opioid consumption and lead to the development of opioid tolerance (38,39).

Opioids lead to immunosuppression via many mechanisms, but not all of them induce the same immune profile. A strong modulating effect has been demonstrated for codeine, methadone, morphine, fentanyl, sufentanil, and remifentanil, and a weak modulating effect has been demonstrated for oxycodone, tramadol, and hydromorphone. The smallest immunomodulatory effect was noted for buprenorphine (40,41). Most publications describe the immunosuppressive effect with respect to the use of morphine. A number of mechanisms for the effects of morphine on immunity have been described. Morphine induces immune suppression by directly regulating adaptive and innate cells, including NK cells, macrophages, mast cells, B cells and T cells. Additionally, morphine administration due to indirect mechanisms connected with central nervous system structures and the HPA axis suppressed NK cell cytotoxicity and lymphoproliferation (42,43). Sacerdote et al. (40) described that chemical structure is a crucial point of immunosuppression: the C_6_ carbonyl substitution, together with the presence of a C_7-_C_8_ single bond, potentiates the antinociceptive effect but minimizes immunosuppression. This chemical construction is characteristic of hydromorphone and oxycodone. Indeed, the single substitution of an allyl on the piperidine ring resulted in a molecule that antagonized the antinociceptive effect but maintained the immunosuppressive effect. Wodehouse et al. (44) presented that morphine significantly increased the interleukin-6 concentration and suppressed NK cell cytotoxicity after gynecological operation, and gene expression profiles suggest that morphine was more immunosuppressive than oxycodone and a nonopioid control analgesic. It has been described that remifentanil-based anesthesia attenuates leucocyte and neutrophil counts during mastectomy (45). Buprenorphine is an opioid that has the smallest immunosuppressive effect, and many authors have suggested considering buprenorphine as a first-line analgesic (46).

Another important clinical issue associated with the opioid-induced immunosuppression is the formation of hyperalgesia. Classically, analgesic doses of morphine activate G molecules of opioid receptor, and inhibite adenylyl cyclase activity. Suprisingly, morphine administration at very low doses induces a parodoxical effect with increase neuronal excitability with creation of hyperalgesia. It was explained that specific activation of non-neural cells in low-doses opioid therapy increases level of pronociceptive stimulation mediators. Inadequate postoperative pain control resulting from the use of inappropriate doses of opioids results in persistent postoperative chronic pain (47). Currently, in order to limit the use of opioids, it is recommended to conduct polypharmacotherapy using multidirectional action of non-opioid analgesics and adjuvants (anti-convulsants, antidepressants, lidocaine, ketamine, magnesium) using their synergistic and additive effects, and to reduce the use of opioid drugs to severe pain, and limit duration of therapy. Moreover, in experimental studies, the natural fatty-acid was recently identified as an endogenous molecule that minimize antinociceptive and immunosuppressive effects of morphine. It was decribed that PEA - N-Palmitoylethanol-amine minimizes the onset of morphine tolerance, decrease activation of glial cells and stopped phenomenon of tolerance, inhibited inflammation and mast cell degranulation (48).

## CONCLUSIONS

The topic of opioid influence on the immune system and carcinogenesis is an important point of acute and chronic pain pharmacotherapy. It should be assumed that this issue is less relevant for patients with cancer at the stage of palliative medicine, when multidirectional medical activities, including pharmacological treatments, are aimed to ensure quality of life. The subject of opioid influence on the development and progression of neoplastic disease is a very new topic, and at the moment, most publications concern the use of these substances during surgical procedures and as perioperative pharmacotherapy. At present, no precise recommendations have been made to limit opioids in the perioperative period in oncologic patients or during any other period of oncologic therapy. However, methods of low-opioid and nonopioid anesthesia have already been developed, and a number of studies indicate that there are possibilities to provide an adequate level of analgesia with proper perioperative safety while limiting the use of opioids. Knowledge about the relationships between long-term opioid intake in noncancer chronic pain pharmacotherapy and the development of cancer is incomplete, but current publications increasingly indicate that patients in these settings are at risk of developing cancer.

In conclusion, knowledge about the immunosuppressive effects and carcinogenesis associated with the use of opioids suggests that there are multifactorial, complex mechanisms underlying the immunological and oxidative stress impacts and carcinogenesis promotion of opioids and that the intensity varies depending on the type of opioid. It should be emphasized that current publications point to limiting the use of opioids in acute and chronic pain treatment. In light of the presented publications, it can be stated that there are valid premises for modifying clinical practice.

## AUTHOR CONTRIBUTIONS

Kosciuczuk U and Lotowska-Cwiklewska AM conceived and designed the study and were responsible for the data acquisition, analysis and interpretation. Knapp P was responsible for the manuscript drafting and critical revision for important intellectual content. All of the authors approved the manuscript final version submitted and all of the authors agree to be accountable for all aspects of the work in ensuring that questions related to the accuracy or integrity of any part of the work are appropriately investigated and resolved.
